# The Ribonucleoprotein Csr Network

**DOI:** 10.3390/ijms141122117

**Published:** 2013-11-08

**Authors:** Ethel Seyll, Laurence Van Melderen

**Affiliations:** Laboratoire de Génétique et Physiologie Bactérienne, IBMM, Faculté des Sciences, Université Libre de Bruxelles (ULB), 12 rue des Professeurs Jeener et Brachet, B-6041 Gosselies, Belgium; E-Mail: eseyll@ulb.ac.be

**Keywords:** Csr network, Rsm network, global regulation, RNA-binding protein, sRNAs, post-transcriptional regulation

## Abstract

Ribonucleoprotein complexes are essential regulatory components in bacteria. In this review, we focus on the carbon storage regulator (Csr) network, which is well conserved in the bacterial world. This regulatory network is composed of the CsrA master regulator, its targets and regulators. CsrA binds to mRNA targets and regulates translation either negatively or positively. Binding to small non-coding RNAs controls activity of this protein. Expression of these regulators is tightly regulated at the level of transcription and stability by various global regulators (RNAses, two-component systems, alarmone). We discuss the implications of these complex regulations in bacterial adaptation.

## 1. Introduction

### 1.1. A Plethora of Ribonucleoprotein Complexes in Bacteria

Ribonucleoprotein complexes are major players in gene expression regulation. The paradigm for ribonucleoprotein complexes is represented by ribosomes. Translation-competent ribosomes are formed by both stable and transient associations between various types of RNAs (rRNAS, tRNAS, mRNAs, tmRNA) and r-proteins (ribosomal proteins) (for review, see [1]). In addition, some r-proteins are involved in feedback regulations. Under specific conditions such as starvation, these proteins negatively regulate their own translation by binding to their encoding mRNA [2].

Other ribonucleoprotein complexes involve the association of small RNAs (sRNAs) with specific proteins. The 6S RNA interacts with σ^70^-containing RNA polymerase and regulates transcription at specific promoters [3]. The 4.5S RNA, a component of the Signal Recognition Particle (SRP), is essential for targeting signal peptide-bearing proteins to inner membrane (for review, see [4]). Another type relies on the association of RNA chaperones, such as Hfq, with regulatory sRNAs to facilitate interaction with their mRNA targets (for review, see [5]). Other RNA chaperones play important roles under specific conditions or in specific systems, such as the cold-shock CspA protein, which destabilizes mRNA structures and thereby facilitates translation at low temperature [6]. Ribonucleases (RNases) also associate transiently with various RNA species. For example, RNaseP and RNaseE are involved in mRNAs and sRNAs maturation, function, and decay (for review, see [7]).

In this review, we focus on a third type of ribonucleoprotein complexes, which rely on the association of a regulatory protein directly with target mRNAs to modulate their translation. We will review the Csr (carbon storage regulator) network in which the CsrA regulatory protein plays a pivotal role. This network is particularly interesting as it involves sRNAs that regulates CsrA activity. We discuss in detail the various ribonucleoprotein complexes involving CsrA as well as the Csr network roles in bacterial physiology.

## 2. The Csr Network

### 2.1. CsrA Is a Global Regulator

The first component of the Csr network was described in 1993 in *E. coli* [8]. In a screen to identify genes involved in glycogen biosynthesis by transposon mutagenesis, the group of Romeo identified the *csrA* gene as encoding a negative regulator of glycogen accumulation. Subsequently, it was shown that CsrA is a 61 amino acid protein that regulates translation by binding to mRNA targets [9,10]. In addition to its implication in glycogen synthesis regulation, CsrA regulates central carbon metabolism. The observation that expression of the *pckA* gene, using a *pckA::lacZ* translational fusion, was increased in the *csrA::kan* mutant provided the first evidence of *csrA* implication in gluconeogenesis regulation [8]. *pckA* encodes phosphoenolpyruvate carboxykinase, a key regulatory enzyme in this pathway. Later on, in a study evaluating glycolysis and gluconeogenesis enzyme activities, gene expression and metabolites, it was inferred that CsrA has a global positive effect on glycolysis and a global negative effect on gluconeogenesis [11]. Accordingly, glucose consumption and extracellular levels of acetate were shown to be reduced in *csrA* depletion conditions [12]. Expression of the *acs* (acetyl-CoA synthetase) and *aceA* (isocitrate lyase) is positively regulated by CsrA [13]. In addition, ATP level is reduced while AMP and ADP levels are increased, indicating a lower energy charge [11]. In a recent paper, global changes upon *csrA* depletion (using overexpression of the sRNA CsrB, see below) were monitored using proteomics and metabolomics approaches [12]. This study confirmed that central carbon metabolism is generally affected in these conditions. Intermediates of glycolysis, phosphoenolpyruvate (PEP), acetyl-CoA and intermediates of the glyoxylate shunt are accumulating. Amino acid and fatty acid metabolisms are also impacted [12]. In species other than *E. coli*, such as *Salmonella*, deletion of *csrA* was also shown to affect metabolism, *i.e.*, maltose transport, ethanolamine utilization, and propanediol metabolism [14].

Thus, a defect in *csrA* drastically alters carbon flux distribution. Effects on gene expression have been shown for a few enzymes, raising the possibility that most of the effects might be consequences of accumulation of specific intermediates that modify enzyme activity and redirect carbon into other metabolites.

In addition to its role in central carbon metabolism, CsrA controls the switch between sessile (biofilm) and planktonic lifestyle [15]. The *csrA::kan* mutation increases adherence [8] while it drastically reduces motility. Several direct regulations mediated by CsrA are involved in this switch. CsrA negatively regulates *pgaABCD* expression which contributes to β-1,6-*N*-acetyl-d-glucosamine (PGA) synthesis [16], an exopolysaccharide involved in adhesion [17]. CsrA positively regulates *flhDC* expression, encoding the flagellar master regulator [18,19]. In addition, the level of c-di-GMP, a signaling molecule that notably controls biofilm formation and motility, is affected in the *csrA::kan* mutant (for review, see [20]). CsrA negatively regulates the expression of seven genes encoding proteins with GGDEF and/or EAL domains (responsible for diguanylate cyclase and phosphodiesterase activities, respectively) [21].

CsrA is also involved in bacterial interactions with animal and plant hosts (for reviews, see [22–24]). For example, CsrA is involved in *Salmonella typhimurium* invasion of epithelial cells and survival inside macrophages [25]. The CsrA homologues in the *Pectobacterium* genus (RsmA) play a crucial role in infection and colonization of host plants [26]. In the opportunistic human pathogen *P. aeruginosa*, RsmA is involved in the control of a wide variety of processes involved in pathogenesis, such as hydrogen cyanide synthesis [27], type IV pili expression [28], quorum sensing [29] as well as type VI and type III secretion systems [28]. In the plant-beneficial root-colonizing strain *P. fluorescens*, RsmA and its homologue RsmE, control synthesis of extracellular antifungal secondary metabolites as well as expression of the exoenzymes AprA and phospholipase C [30,31].

As described above, the *csrA::kan* mutant shows drastic phenotypes although this mutant does not display any growth defect. It is able to grow on rich medium (LB) as well as on minimal medium supplemented with gluconeogenic or glycolytic carbon sources [8]. The *kan* transposon is inserted at codon 51 in the *csrA* gene [8], leaving the possibility to produce a truncated CsrA protein that is still partially active [19,32]. This mutant is the basis for CsrA biological roles characterization and is used in most of the studies performed on the *E. coli* model. In *Salmonella*, a *csrA* deletion mutant was constructed and severe growth defect and selection of suppression mutants with time were described [33]. Similar phenotypes were observed recently in an *E. coli* uropathogenic strain [34]. *csrA* deletion mutants were also constructed in K-12 and uropathogenic *E. coli* isolates in our group and we observed similar growth defect and suppressor selection [35]. On the contrary to our earlier published data, deletion of the *glgCAP* operon in the K-12 or uropathogenic strains does not improve growth [32]. This suggests that suppressor mutants were also picked up in the K-12 *ΔcsrA ΔglgCAP* mutant [35].

### 2.2. Building up the Csr Network

In the years following CsrA identification, additional components of the Csr network were identified, notably sRNAs and proteins regulating either its activity or its expression. CsrA acts as a dimer [10,36] and forms ribonucleoprotein complexes with two sRNAs, CsrB [37], and CsrC [38]. Interaction of CsrA with these regulators leads to CsrA sequestration and inhibition of activity. These sRNAs are structurally similar although they carry a variable number of CsrA binding sites [39,40]. CsrB is 366 nt long containing 22 putative CsrA binding sites. It was shown that this sRNA is able to bind up to 18 CsrA dimers (see below), as shown by molecular weight estimation (256 kDa) [37]. The CsrC sRNA (242 nt long) contains 14 putative CsrA binding sites and is able to bind nine CsrA dimers [38].

Experimental data indicate that up to one-third of CsrA may be bound to the CsrB sRNA [41]. As CsrA affinity for these sRNAs is higher than that for mRNA targets and its concentration is higher than CsrA-target dissociation constant [41,42], it is inferred that CsrB and CsrC levels determine the concentration of “active” CsrA. Regulation of expression of these sRNAs is thus crucial to control CsrA activity. A negative feedback loop is involved in this regulation. CsrA indirectly activates *csrB* and *csrC* transcription through the BarA/UvrY two-component system (TCS) [38,41,43]. The BarA-associated response regulator, UvrY, activates transcription of the regulatory sRNAs [38,43–45]. The Csr network is composed of additional feedback loops *i.e.*, CsrA directly represses its own translation, while it indirectly activates its own transcription by an unknown mechanism [46]. These regulatory loops provide a rapid mechanism to reduce *csrA* expression when concentration of free CsrA reaches critical levels. The physiological signals that trigger the Csr network through the membrane-bound BarA sensor kinase have been identified. Interestingly, signals are products of carbon metabolism such as formate, acetate, other short-chain fatty acids, and Krebs cycle intermediates, which correlates with the crucial roles played by CsrA in central carbon metabolism [47,48].

Another important player in the Csr network is the CsrD protein. CsrD is predicted to be a membrane-bound protein containing degenerate GGDEF and EAL domains [49]. It controls the decay of CsrB and CsrC in an RNase E-dependent manner and in a c-di-GMP-independent manner, although both domains appear to be necessary for CsrD activity [19,49]. The “destabilizing” activity of CsrD on CsrB and CsrC has a positive effect on CsrA activity [49]. In addition, CsrA negatively controls *csrD* expression, which indicates an additional feedback loop in the Csr network [21,49,50]. It is not excluded that other types of regulation might exist within the Csr network. For example, in *Bacillus subtilis*, CsrA activity is regulated by direct interaction with the FliW protein, rather than sRNAs, in order to regulate motility [51].

### 2.3. Expanding the Csr Network

In the previous section, we described the basis of the Csr network in *E. coli*. Orthologues of *csrA* and *csr* regulatory elements are detected throughout the bacterial world. Thus, Csr constitutes a conserved global regulatory network, also called Rsm (repressor of secondary metabolites) network in specific species, such as *Pectobacterium carotovorum* and in the *Pseudomonas* genus. Interestingly, some species encode multiple *csrA* homologues. While a single copy of the *csrA* gene is present in enteric bacteria (e.g., *E. coli*, *Salmonella enterica*, and *Pectobacterium* spp.) and *P. aeruginosa*, *in silico* analyses identified up to five homologues (*rsmA* and *rsmE)* in specific isolates of *P. fluorescens*, *P. putida* KT2440, and *P. syringae* [52]. The number of Csr sRNAs homologues can also vary, e.g., *Vibrio cholerae* and *Pseudomonas fluorescens* have been shown to contain three of them [53,54].

**Figure 1:**
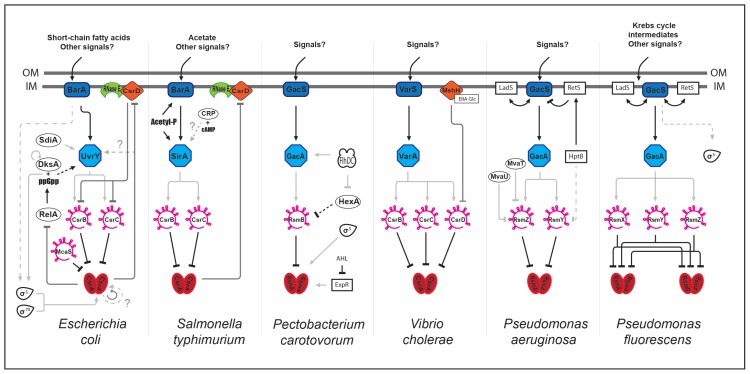
Interactions between the Csr network and global regulatory circuitries. Networks of *Escherichia coli*, *Salmonella typhimurium*, *Pectobacterium carotovorum*, *Virbio cholerae*, *Pseudomonas aeruginosa*, and *Pseudomonas fluorescens* are represented. The CsrA/RsmA/RsmE master regulator is negatively regulated by the Csr/Rsm-sRNAs, which are positively regulated by the BarA/UvrY TCS or its orthologues. Light grey, dark grey, and black lines represent transcriptional, post-transcriptional and post-translational regulations, respectively. Solid and dashed lines represent direct and indirect regulation, respectively. For details, see in the text.

As described above, CsrA regulates different processes. In addition to these direct and specific regulations, the Csr partners (proteins and sRNAs) interact with other global regulatory networks. How these interactions might influence group behavior such as quorum sensing, motility and biofilm formation remain vague, although several studies have highlighted tight connections between these different pathways (Figure 1).

Some of these connections take place via CsrA. In *E. coli*, CsrA modulates the ppGpp alarmone level by directly regulating *relA* expression and thereby linking the Csr network to stringent response [55]. In turn, ppGpp and the DksA transcription factor regulate *csrB* and *csrC* expression via an unknown mechanism. In *P. carotovorum*, CsrA is regulated by another regulatory cascade involving a putative *N*-acyl homoserine lactone receptor (ExpR), thereby linking quorum sensing and Csr [56]. The connection between these two pathways is also described in *E. coli* although at another level and through different actors. In this case, the SdiA protein, a LuxR homologue, activates *uvrY* expression at the transcriptional level, thereby affecting *csrB* expression [43].

Interestingly, the Csr network is connected to metabolism through a CsrD orthologue (MshH) in *V. cholerae* [57]. MshH directly interacts with the EIIA^Glc^ enzyme from the PTS system (phosphoenolpyruvate:carbohydrate phosphotransferase system) to positively control biofilm formation in a CsrA-independent manner. Connections between the Csr network and metabolism are also described in *S. typhimurium* through the BarA/SirA TCS. The UvrY orthologue (SirA) is positively regulated by catabolite repression or directly phosphorylated by acetyl-phosphate, a product generated by acetate metabolism [58–61]. Connections between the Csr network and motility are also mediated through the UvrY orthologue in specific species, such as in *P. carotovorum*. Expression of *gacA* (homolog to *uvrY*) is positively regulated by FlhDC [62]. This regulator acts positively on the *csrB* orthologue *rsmB* via HexA, a LysR-like regulator known to control exoenzymes production, further linking the Csr network to virulence [62,63].

In *P. aeruginosa* and *P. fluorescens*, another layer of complexity is added. Cross-regulation of GacS (BarA orthologue) by the LadS and RetS hybrid sensor kinases (containing both the sensor kinase and response regulator activities) have been shown. These two regulators are involved in pathogenesis and act in an opposite manner since RetS promotes acute infections whereas LadS promotes a chronic state [64–66]. This allows the integration of multiple signals and lead to a fine-tuned response.

Interestingly, other sRNAs might be involved in the Csr network to control CsrA activity such as McaS (multicellular adhesive) sRNA in *E. coli*, which possess a dual function. It controls biofilm formation by classical base-pairing with mRNA targets and additionally, by sequestrating CsrA [67].

In *E. coli* and *P. carotovorum*, the Csr network is connected to the general stress response via the RpoS sigma factor, highlighting further complex interactions between pivotal pathways [46,68].

### 2.4. Are the Regulatory Csr/Rsm sRNAs Redundant?

The question of Csr/Rsm sRNAs redundancy was approached in different species. In general, it appears that these sRNAs are redundant. For examples, in *P. fluorescens*, the single *rsmY* or *rsmZ* mutants are not affected for extracellular enzymes production while the double mutant is unable to produce these virulence factors [69]. In *S. typhimurium*, mutants of both *csrB* and *csrC* genes are required to obtain phenotypes similar to that of a CsrA overexpression strain [70]. Furthermore, in *E. coli*, expression of one of the Csr sRNA is increased in mutants deleted of the other one, suggesting a compensatory mechanism [38]. Although these regulatory sRNAs are functionally and structurally related and regulated by the BarA/UvrY TCS or its homologues, they present specific features. One important functional difference resides in their capacity to bind a variable number of CsrA molecules. As a result, CsrC shows lower affinity for CsrA than CsrB [38]. In addition, molecular mechanisms regulating CsrB and CsrC stability in *E. coli* are different, although both require RNAseE. CsrB degradation requires CsrD and PNPase (polynucleotide phosphorylase, an exonuclease that is also part of the degradosome (for review, see [71]) while that of CsrC is independent of these two enzymes [49]. Additional differences between CsrB and CsrC expression and/or stability are observed in other species. Although expression of CsrB and CsrC in *Y. pseudotuberculosis* is regulated by UvrY, catabolite repression contributes to differential expression of the sRNAs with CsrB being up-regulated in a *crp* (cAMP receptor protein) mutant and CsrC down-regulated [72]. This observation provides an additional example of connection between carbon metabolism and the Csr network. In *P. aeruginosa*, global regulators belonging to the H-NS family (MvaT and MvaU) regulate expression of the *rsmZ* sRNA but not that of *rsmY* (Figure 1) [73]. In addition, RetS and the HptB protein involved in phosphotransfer cascade protein, control *rsmY* expression, whereas *rsmZ* expression is exclusively controlled by RetS [74]. Regarding sRNAs stability, data indicate that differential regulation may also occur. In *P. aeruginosa*, Hfq binds and stabilizes RsmY [75]. In *S. typhimurium*, CsrA positively regulates CsrC half-life, suggesting that CsrA binding might lead to CsrC stabilization [70].

Difference in regulation of sRNA levels may provide clues to understand the benefit of having two or more seemingly redundant sRNAs. This may allow more possibilities for integrating various signals, leading to efficient and precise regulatory responses via gene dosage effect [73].

## 3. The CsrA-Containing Ribonucleoprotein Complexes

### 3.1. Structural Information

**Figure 2:**
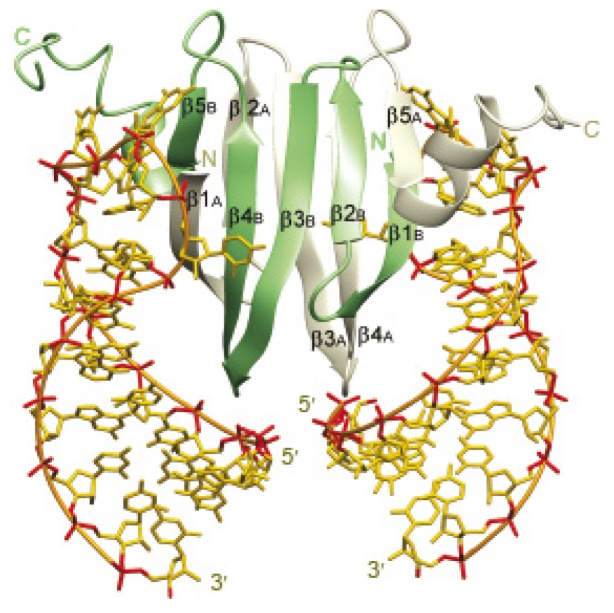
Structure of the RsmE-RNA complex of *P. fluorescens*. Solution structure of the 2:2 complex of RsmE with 20-nucleotide *hcnA* sequence. Protein ribbons belonging to each monomer are shown in green and grey. Heavy atoms of the two RNAs are shown in yellow (carbon and nitrogen) and red (oxygen and phosphorus). The linking phosphates are shown in orange. From [42].

The CsrA protein is unrelated to typical regulators in terms of amino acid sequence but contains a KH motif (single-strand RNA binding domain) [76]. NMR-based structure shows that CsrA is composed of 5 β-strands and a short α-helix followed by an unstructured carboxy-terminal region (Figure 2) [36]. The functional CsrA dimer is formed by interdigitation of two CsrA monomers, resulting in a hydrophobic core composed of 10 β-strands and two wing-like α-helices, forming a barrel-like structure [77]. Using comprehensive alanine-scanning mutagenesis, two critical regions for regulation and RNA binding were identified [78]. These two RNA-binding surfaces are located within the first and last β-strands, which lie parallel to each other on opposite sides of the dimer and form two positively charged regions [78].

Several amino acids residues are involved in RNA-binding, with R44 being the most important, and less importantly Arg7 and Ile47 [78]. CsrA preferentially binds GGA motifs (see below) located in single-strand regions of short hairpin structures [42,78,79]. Sequence-specific recognition of this motif is mostly mediated by carbonyl oxygen and amide groups of the main chain, indicating that CsrA-fold itself is responsible for RNA binding specificity [42].

### 3.2. Mechanism of Action of CsrA on mRNAs Targets

CsrA regulates translation of its target mRNAs, either positively or negatively. mRNA targets contain a variable number of CsrA binding sites. In general, they are located in the untranslated leader sequence and overlap the Shine-Dalgarno (SD) sequence. The CsrA-binding consensus sequence closely matches the canonical AAGGAGGU SD sequence [42]. The number of CsrA binding sites may vary from 1 to 6, depending on the target. For example, 4 and 6 CsrA binding sites have been identified on the *glgCAP* and *pgaABCD* mRNA targets, respectively [17,80]. In the case of negative regulation, binding of CsrA prevents translation initiation and generally leads to mRNA degradation [9]. Some variations in the molecular mechanisms have been shown. As an example, negative regulation of *sdiA* occurs by CsrA binding to two sites located within the early coding region of the *sdiA* mRNA, without binding to or occluding the SD sequence [81].

Positive regulation appears to be less common. Up to now, two positively regulated targets have been described. In the case of *flhDC*, two binding sites are located in the untranslated leader region of the transcript. CsrA binding to these sites stabilizes the transcript by inhibiting the 5′-end degradation mediated by RNaseE [18,19]. The second example involves the regulation of the *moaA* gene, which is involved in molybdenum cofactor (MOCO) synthesis. MOCO serves as a redox center in enzymes of anaerobic metabolism. The *moa* mRNA untranslated leader sequence contains two CsrA-binding sites and constitutes a MOCO-sensing riboswitch. This is the first example of a riboswitch aptamer that interacts with two regulatory factors, a low-molecular-weight ligand and an RNA binding protein [82].

### 3.3. Deeper Understanding of the Ribonucleoprotein Complexes Formed by CsrA

Although considerable sequence variation exists among the known CsrA binding sites, a GGA motif was identified as a highly conserved and essential element [79]. This was confirmed by studies using the SELEX method (systematic evolution of ligands by exponential enrichment). The SELEX-derived consensus was determined as RUACARGGAUGU (R being a pyrimidine and the underlined GGA motif being essential) [79]. As described above, the GGA motif is often located in single-strand loops of predicted short RNA hairpins [40]. A single-strand structure appears to be mandatory for regulation since several sRNAs (GcvB, RprA and OmrA) that carry potential CsrA-binding sites with GGA motifs located in double-strand structures (secondary structures of these sRNAs were predicted using Mfold [53], data not shown) do not regulate nor bind CsrA [67]. Spacing distance between CsrA binding sites appears to be important for CsrA-target complex stability [83]. The optimal distance is 18 nt although CsrA dimers are able to bind to two target sites separated by 10 to 63 nt. In CsrB, the 22 potential CsrA target sites are separated by an average of 12.25 nt. This suggests that a CsrA dimer would preferentially bridge non-adjacent sites (*i.e.*, separated by at least 18 nt), giving rise to an energetically stable globular complex. Dubey *et al*. [79] proposed a model for CsrA binding. Translation repression mediated by CsrA would involve initial binding to a high affinity site located within a hairpin loop upstream of the SD sequence. This first binding would then allow the free RNA-binding surface of CsrA to interact with downstream low-affinity sites overlapping the SD sequence. These two successive binding events would result in formation of a repression loop blocking ribosomal binding for translation initiation [83].

## 4. General Conclusions

Ribonucleoprotein complexes control multiple pathways and may act at different levels of gene expression regulation. Regulation by ribonucleoprotein complexes comes into different flavors, with for instance, structural proteins acting also as post-transcriptional regulators such as RodZ, a cytoskeletal protein, that post-transcriptionally regulates expression of type III secretion system in *Shigella* [84].

In this review, we have described the Csr network with a special focus on the interactions between the CsrA key regulator and various species of RNAs *i.e.*, mRNA targets and regulatory sRNAs. sRNAs regulate the key regulator availability by sequestering it, thereby limiting the cellular concentration of “active” CsrA. One might propose that, like the McaS sRNA [67], these sRNAs have additional CsrA-independent functions. The fact that Hfq binds to RsmY [75] may indicate that this sRNA possesses regulatory activity involving mRNA-base pairing, as shown for McaS [67].

As exemplified in this review, ribonucleoprotein complexes connect multiple regulatory networks to coordinate gene expression and lead to adaptation.
